# Spatially resolved emulated annual temperature projections for overshoot pathways

**DOI:** 10.1038/s41597-024-04122-1

**Published:** 2024-11-21

**Authors:** Jonas Schwaab, Mathias Hauser, Robin D. Lamboll, Lea Beusch, Lukas Gudmundsson, Yann Quilcaille, Quentin Lejeune, Sarah Schöngart, Carl-Friedrich Schleussner, Shruti Nath, Joeri Rogelj, Zebedee Nicholls, Sonia I. Seneviratne

**Affiliations:** 1 Institute for Atmospheric and Climate Science, Department of Environmental Systems Science, ETH Zurich, Zurich, Switzerland; 2 WSL Institute for Snow and Avalanche Research (SLF), Davos Dorf, Switzerland; 3 Centre for Environmental Policy, Imperial College London, London, UK; 4 Federal Office of Meteorology and Climatology MeteoSwiss, Zurich, Switzerland; 5 Climate Analytics, Berlin, Germany; 6 IRIThesys, HU Berlin, Friedrichstrase 191, 10117 Berlin, Germany; 7 International Institute for Applied Systems Analysis (IIASA), Laxenburg, Austria; 8 Geography Department and IRITHESys Institute, Humboldt-Universität zu Berlin, Berlin, Germany; 9 Grantham Institute for Climate Change and the Environment, Imperial College London, London, UK; 10 Energy, Climate and Environment Program, International Institute for Applied Systems Analysis, Laxenburg, Austria; 11 Climate Resource, Fitzroy, Victoria Australia; 12 School of Geography, Earth and Atmospheric Sciences, The University of Melbourne, Melbourne, Australia

**Keywords:** Climate and Earth system modelling, Governance, Climate-change mitigation

## Abstract

Due to insufficient climate action over the past decade, it is increasingly likely that 1.5 °C of global warming will be exceeded – at least temporarily – in the 21^st^ century. Such a temporary temperature overshoot carries additional climate risks which are poorly understood. Earth System Model climate projections are only available for a very limited number of overshoot pathways, thereby preventing comprehensive analysis of their impacts. Here, we address this issue by presenting a novel dataset of spatially resolved emulated annual temperature projections for different overshoot pathways. The dataset was created using the FaIR and MESMER emulators. First, FaIR was employed to translate ten different emission scenarios, including seven that are characterised by overshoot, into a large ensemble of forced global mean temperatures. These global mean temperatures were then converted into stochastic ensembles of local annual temperature fields using MESMER. To ensure an optimal tradeoff between accurate characterization of the ensemble spread and storage requirements for large ensembles, this procedure was accompanied by testing the sensitivity of sample quantiles to different ensemble sizes. The resulting dataset offers the unique opportunity to study local and regional climate change impacts of a range of overshoot scenarios, including the timing and magnitude of temperature thresholds exceedance.

## Background & Summary

Mitigation pathways that aim to meet the temperature goal of the Paris Agreement to hold global warming well below 2 °C above pre-industrial levels and to pursue limiting it to 1.5 °C (UNFCCC 2015), include scenarios that temporarily exceed 1.5 °C of global warming. Such overshoot pathways will exacerbate adverse impacts during and beyond the period of overshoot^1–4^. Crossing thresholds in global mean temperature (GMT) – even temporarily – can have particularly severe consequences for vulnerable regions and their adaptation limits^5–7^. Indeed, increases in regional climate extremes and impacts associated with overshoots above 1.5 °C can be substantial even for half a degree of global warming, or even a tenth of a degree of global warming^8^, i.e., with peak temperatures within 1.6–2 °C of global warming or higher. The impacts of overshoot scenarios depend on the timing and magnitude of the GMT threshold crossing^7^. However, to date, spatially explicit climate data for overshoot scenarios are scarce. To allow for a comprehensive analysis of overshoot pathways and their local and regional impacts, we present a dataset containing local annual temperature fields of ten different emission scenarios of which seven are characterized by overshoot. The data is produced relying on computationally efficient and well-validated climate model emulators^9–11^.

Climate emulators (CEs) encompass a variety of reduced-complexity climate models that reproduce parts of the behavior of Earth System Models (ESMs) to represent key features of the climate system^12^. CEs are computationally efficient and can therefore be used when running ESMs is computationally too demanding^13^. Prominent use cases are the production and exploration of large sets of emission scenarios and of large probabilistic ensembles^14–16^.

**Fig. 1:**
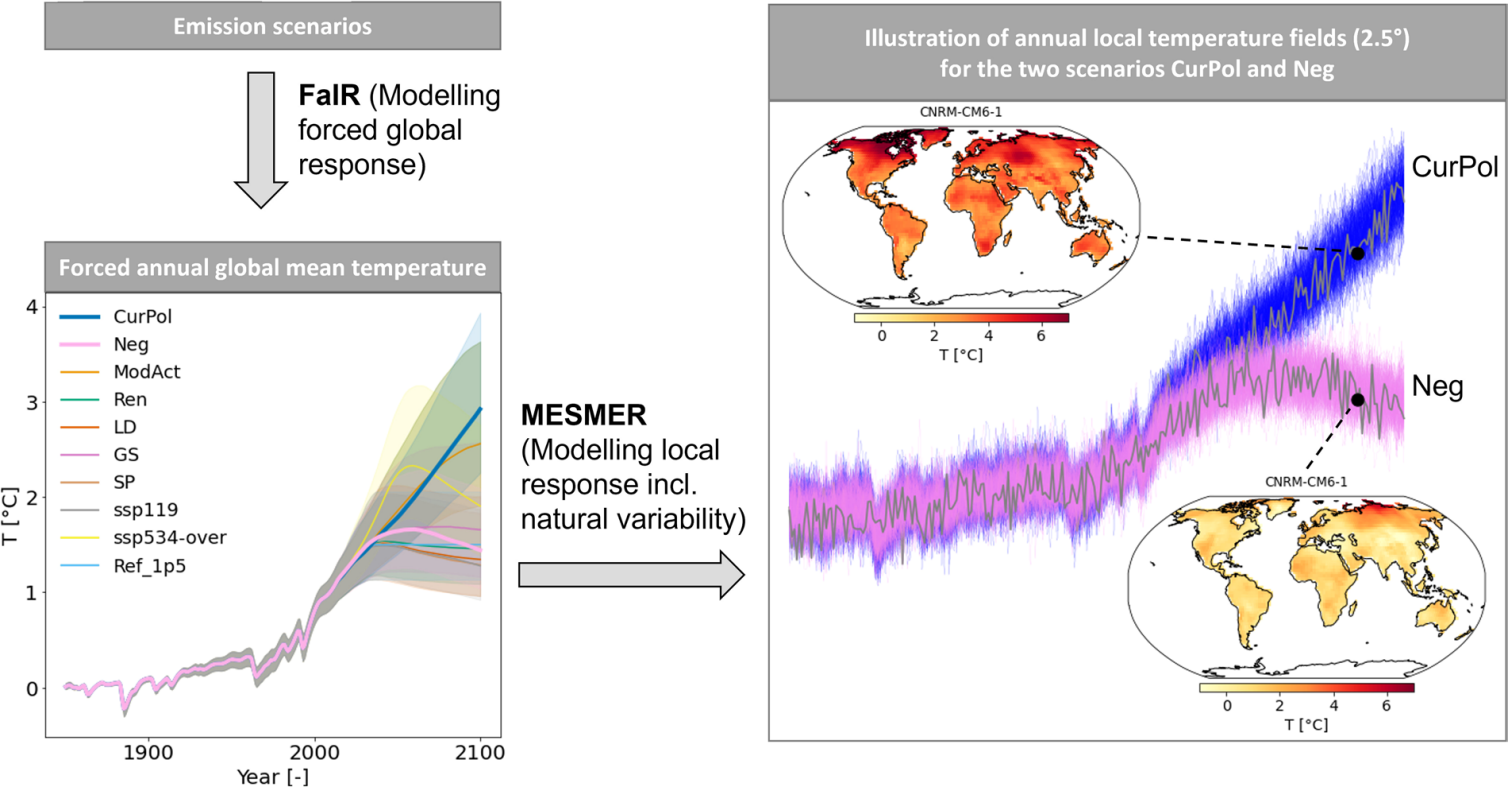
Illustration of the FaIR – MESMER emulator chain. The FaIR emulator is used to translate emission pathways into global mean temperature estimates. These are then used to force MESMER to emulate spatially explicit annual mean temperatures.

Several CEs focus on estimating the climate response to different emission scenarios for large regions or the entire globe^13^. Other emulators focus on generating spatially resolved climate fields at the spatial resolution of ESMs^10,17–19^. For example, the regional MESMER (Modular Earth System Model Emulator with spatially Resolved output) emulator is designed to emulate the forced climate response and natural variability at grid-level scales^10^, which makes it an ideal candidate to generate large emulated climate ensembles to assess their link to socio-economic and environmental impacts of climate change. However, MESMER is not directly driven by the atmosphere’s radiative forcing, which is impacted by human emissions. It instead calculates spatially resolved temperatures based on GMT trajectories. Therefore, deriving spatially resolved temperatures based on different emission scenarios, requires combining MESMER with an emulator that translates human emissions into global mean temperatures (Fig. 1, Beusch, *et al*.^11^).

Here, we rely on GMT trajectories generated by the Finite amplitude Impulse Response (FaIR) model^9^ based on 10 different emission scenarios^20^ and use them to create an ensemble of MESMER emulations (Fig. 1). We largely follow the framework presented in Beusch, *et al*.^11^. The resulting dataset^21^ is the first of its kind in that it (i) contains annual local land temperature fields for a variety of different overshoot scenarios for which no ESM climate simulations are available and (ii) consists of a large number of stochastic realizations (=MESMER emulations) that allow for an exploration of the uncertainty due to natural climate variability.

## Methods

This section is structured as follows: First, we introduce the FaIR and MESMER emulators and their most important settings (Section 2.1), second, we describe how to reduce the number of emulations in the ensemble while preserving all its main characteristics (Section 2.2). The latter step is necessary as, for each emission pathway, FaIR can produce many stochastic realizations, and from each FaIR realization numerous MESMER emulations can be derived. This can lead to an exponential increase in the number of emulations when coupling the two emulators. A large number of emulations is advantageous because it allows for a more precise analysis of the properties and uncertainties inherent to a large ensemble of emulations and as both emulators have negligible computational cost, such an ensemble can be generated very easily. However, the main limitation in having an enormous number of emulations is the huge amount of storage and post-processing time required. Thus, we provide an overview of how the ensemble size of the emulations can be reduced while balancing the trade-off between adequate representation of the ensemble and data storage requirements. The outlined methodology is generic and could be used for any combination of globally aggregated^9,22,23^ and spatially resolved emulators^10,17–19^.

### FaIR and MESMER

The simple climate model FaIR (version 1.6.4, Smith, *et al*.^9^) was used to generate a range of forced annual GMT trajectories based on ten different emission scenarios through 2100 (Fig. 1). These data are openly available (https://zenodo.org/records/6833278#.YtBTkXbMJPY).Details describing how the data was produced can be found in Lamboll, *et al*.^20^ and are briefly summarized here. The ten scenarios are mainly derived from the existing literature. The majority (seven out of ten) are based on the IPCC’s Illustrative Mitigation Pathway (IMP) scenarios, two are based on extensions of the Shared Socioeconomic Pathways (SSPs) and one is an idealized temperature-based scenario (Table S1). Because the data for these different scenarios is usually not complete (i.e., emissions other than CO_2_ are often unavailable), the Silicone module^24^ has been used to infer missing emission data required to run FaIR. The ten scenarios are described in Lamboll, *et al*.^20^ and in Table S2. In short, the scenarios are CurPol (Current Policy pathway), ModAct (Moderate Action pathway), GS (Gradual Strengthening pathway), Neg (Negative emissions pathway), Ren (Renewable pathway), LD (Demand-Limiting pathway), SP (Shifting Pathways), SSP1-1.9, SSP5-3.4-OS (SSP5.3.4 overshoot) and Ref1.5 C (following LD until temperature reaches 1.5 °C and afterwards holding temperature constant).

The MESMER emulator was used to generate spatially explicit annual temperature fields based on the generated FaIR GMT trajectories (Fig. 1, Schwaab, *et al*.^21^). To couple MESMER and FaIR we mainly relied on mesmer-openscmrunner (https://github.com/MESMER-group/mesmer-openscmrunner). The emulator was introduced to emulate multi-ESM initial-condition ensembles to create spatially resolved annual mean temperature anomalies and to emulate multiple emission scenarios^11^. The local temperature anomalies are modeled as a function of the GMT change and by a spatiotemporally correlated noise term. We rely on MESMER version 0.8.3, which is described in detail in Beusch, *et al*.^11^ and includes all trained MESMER parameters that are necessary to perform the emulations (https://zenodo.org/records/5802054). Version 0.8.3 includes the option to model the local forced response as a non-linear function of GMT change and of ocean heat uptake. However, we used the default approach of modeling the local forced response as a linear function of GMT, which has been shown to produce emulations of similar quality^11^. MESMER was calibrated on a set of 25 ESMs (Table 1) from the sixth phase of the Coupled Model Intercomparison Project (CMIP6, Eyring, *et al*.^25^, https://esgf-node.llnl.gov/projects/esgf-llnl/).

**Table 1:** Overview of the data structure.

Emission scenarios/Illustrative Mitigation Pathways (IMPs)	**CurPol** (Current Policies), **Moderate Action** (ModAct), **GS** (Gradual Strenghtening of Current Policies), **SP** (Shifting Pathways), **LD** (Low Demand), **Neg** (Extensive Use of Net Negative Emissions), **Ren** (Renewables), **SSP1-1.9,** **SSP5.3.4-Os** (Overshoot), **Ref_**1p5 (see Table S1/S2 and Lamboll, *et al*.^20^ for more details)
Run id	Id specifying each FaIR ensemble member (2237 per emission scenario)
ESMs	ESM used for MESMER calibration (ACCESS-CM2, ACCESS-ESM1-5, AWI-CM-1-1-MR, CanESM5, CESM2, CESM2-WACCM, CMCC-CM2-SR5, CNRM-CM6-1, CNRM-CM6-1-HR, CNRM-ESM2-1, E3SM-1-1, FGOALS-f3-L, FGOALS-g3, FIO-ESM-2-0, HadGEM3-GC31-LL, HadGEM3-GC31-MM, IPSL-CM6A-LR, MCM-UA-1-0, MPI-ESM1-2-HR, MPI-ESM1-2-LR, MRI-ESM2-0, NESM3, NorESM2-LM, NorESM2-MM, UKESM1-0-LL)
Realization	Id specifying each stochastic MESMER realization (10 per ESM-specific calibration)

In addition to producing temperature anomalies, we provide temperature data of the reference period (1850–1900) for each ESM, which can be used to calculate future changes in absolute temperature. This required two small modifications to the approach outlined by Beusch, *et al*.^11^. First, the temperature averages over the reference period were averaged over all ensemble members as opposed to calculating them for each initial-condition ensemble member individually. The averages are needed to compute temperature anomalies. Second, the absolute temperatures for each reference period were saved as an additional output variable of MESMER.

### Sampling strategies for reducing the number of emulations

Our aim is to reduce the number of emulations to save storage space and allow for fast processing of the data, but to produce enough emulations to facilitate accurate estimates of the distribution of climate variables in future climate (i.e., overshoot scenarios). We introduce the following approach for estimating how well different ensemble sizes measure the location and dispersion of temperatures estimated using the emulator chain: First, we calculate quantiles for varying sample sizes (i.e. number of emulations) and for different strategies of how to select a sample. Second, we show mean absolute errors (MAE) and mean bias errors (MBE) between quantiles that are computed for different sample sizes and sample selection strategies.

**Fig. 2:**
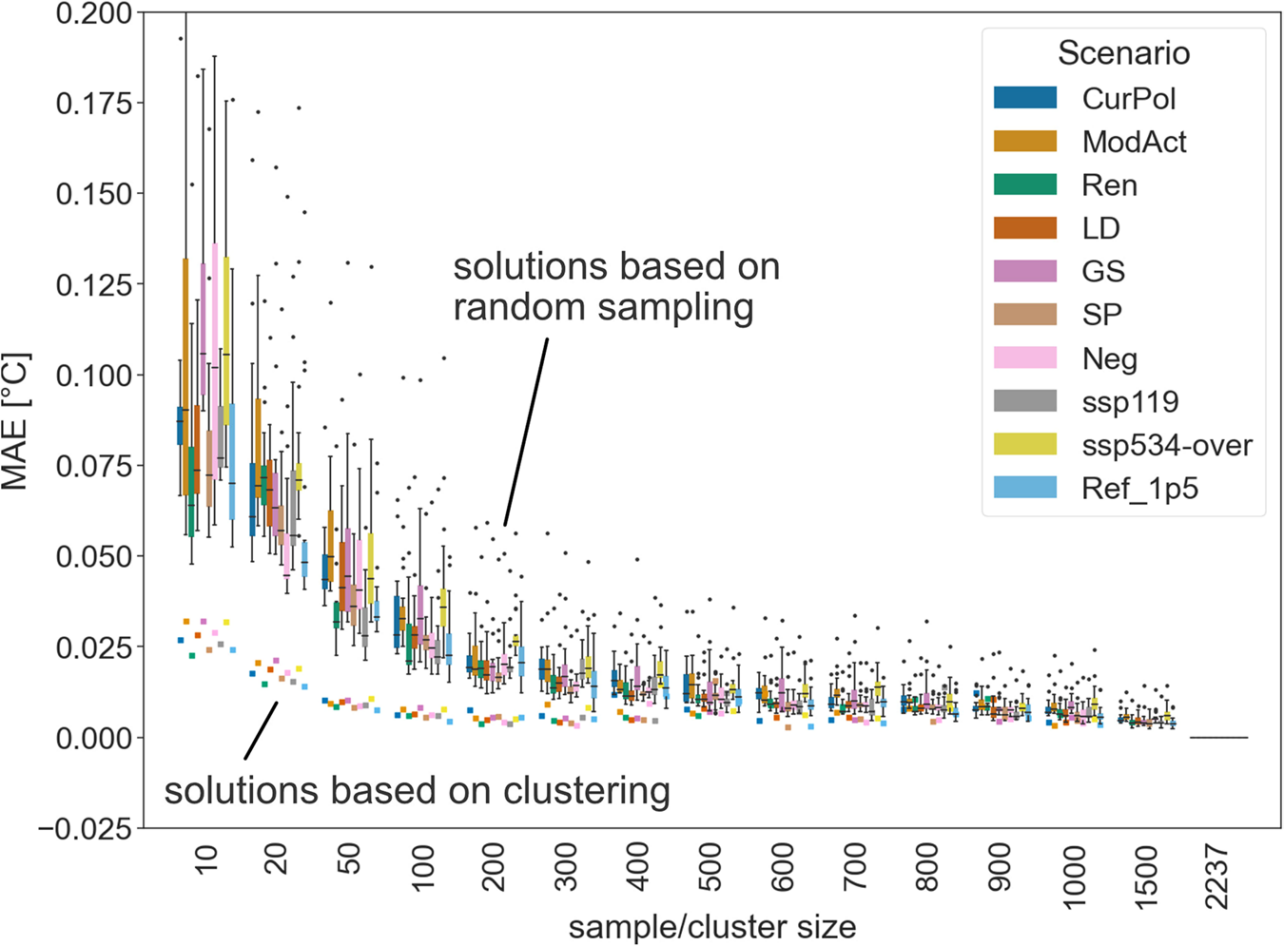
MAEs between quantiles calculated for repeated random sampling of FaIR GMT trajectories (boxplots) and MAEs calculated when selecting FaIR GMT trajectories based on balanced clustering (squared dots). Reference quantiles were calculated using all 2237 FaIR trajectories.

First, we show the MAE between time series quantiles calculated when taking all 2237 FaIR GMT trajectories into account and quantiles calculated when varying the number of randomly selected GMT trajectories between 10 and 1500 (an example is shown in Figure S1):

1 $${{MAE}}_{{sz},{sc}}=\frac{1}{{mn}}\mathop{\sum }\limits_{q=1}^{m}\mathop{\sum }\limits_{t=1}^{n}\left|{T}_{t,q,2237,{sc}}-{T}_{t,q,{sz},{sc}}\right|$$

where MAEs are calculated for each sample size sz and scenario sc based on global mean temperatures T for each quantile q and each year t. For each number of selected GMT trajectories (i.e. each sample size), the random draws were repeated 20 times to explore the sampling properties of the MAEs. We only calculated the MAEs for the years 2010 to 2100, because we are mainly interested in future projections and the FaIR GMT trajectories between 1850 and 2010 are very similar. The results show that there is a strong decrease in MAE as the sample size increases from 10 to 100 (Fig. 2), and that the error for each random sample with 100 emulations generally lies below 0.1 °C. Further increasing the sample size (from 100 to 1500) leads to small reductions in MAE.

In addition to the random sampling of FaIR GMT trajectories, we select FaIR trajectories based on constrained k-means clustering. Relying on constrained k-means clustering as described in Bradley, *et al*.^26^ and implemented by Levy-Kramer *et al*. (https://github.com/joshlk/k-means-constrained), allows us to produce balanced clusters (Figure S2). This means that all clusters are approximately the same size (the maximum difference in cluster size was 1). After clustering, the center of each cluster was matched with a GMT trajectory that was closest to the cluster center. As a criterion for matching the cluster centers with a corresponding trajectory, we calculated MAEs between each GMT trajectory and the cluster centers and selected the trajectory with the smallest MAE. The number of clusters ( = number of cluster centers) was chosen to be aligned with the size of the random samples, but to reduce computational costs the maximum number of clusters was set to 1000. Relying on constrained k-means clustering, it was possible to substantially reduce MAE values in comparison to randomly selected samples (Fig. 2). For example, similar MAEs can be achieved when selecting 100 GMT trajectories based on the clustering approach and 1000 trajectories based on random sampling.

**Fig. 3:**
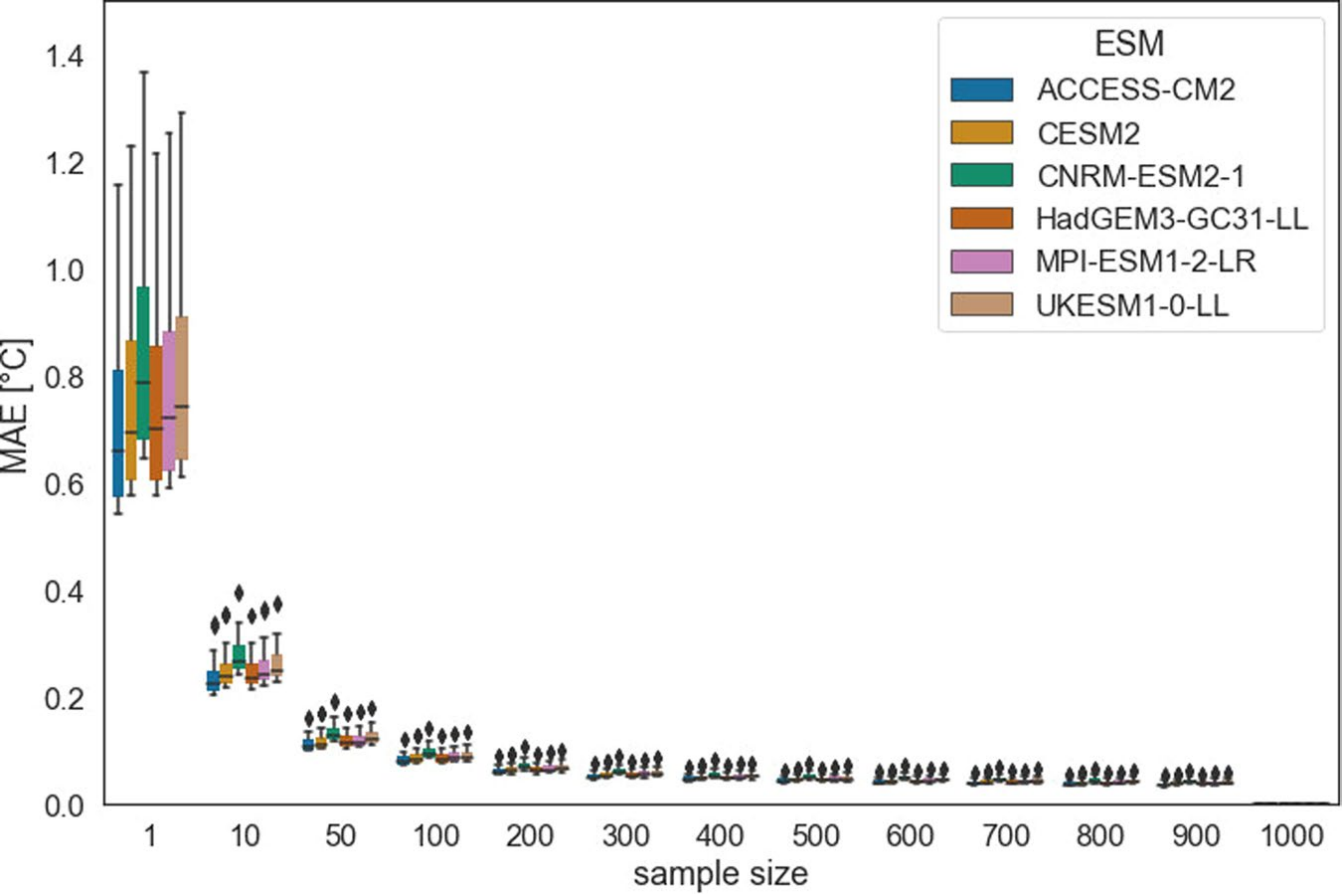
MAEs calculated between quantiles of varying numbers of MESMER realizations for a single FaIR GMT trajectory and for each grid point (e.g. the MAE for sample size 100 indicates the MAE calculated between quantiles estimated based on 1000 realizations and quantiles based on 100 emulations). The comparison of quantiles is shown for 6 randomly selected ESMs (the results for all ESMs are shown in Figure S3).

**Fig. 4:**
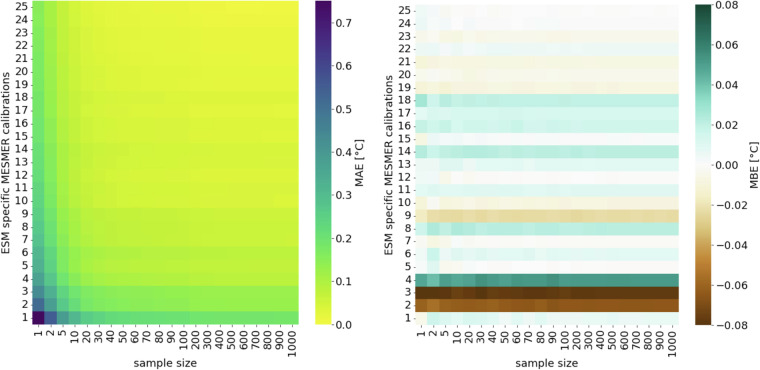
(**a**) MAEs between quantiles calculated for a varying number of ESMs and stochastic realizations per ESM with MESMER (for a single FaIR GMT trajectory). (**b**) MBEs between quantiles calculated for a varying number of ESMs and stochastic realizations per ESM with MESMER (for a single FaIR GMT trajectory).

Second, we generated a varying number of stochastic MESMER realizations (between 1 and 1000) for a single FaIR GMT trajectory and for each individual ESM-specific calibration of MESMER. To illustrate the results, we selected a random FaIR GMT trajectory from the “ssp534-over” scenario. We then computed quantiles for each grid-point and for each ESM-specific MESMER calibration, including 1000 stochastic realizations (as a reference) and for a smaller number of realizations. Again, we observe a steep decrease in MAEs averaged over all grid points with larger sample size (Fig. 3, Figure S3). We also calculated quantiles for each grid point for emulations that were created for varying numbers of ESM-specific MESMER calibrations and varying numbers of stochastic MESMER realizations per ESM-specific MESMER calibration simultaneously. This helped to get a more comprehensive picture of how MAE and MBE change, which were calculated as:

2 $${{MAE}}_{{sz}}=\frac{1}{{mnp}}\mathop{\sum }\limits_{q=1}^{m}\mathop{\sum }\limits_{t=1}^{n}\mathop{\sum }\limits_{s=1}^{p}{\omega }_{s}^{{\prime} }\left|{T}_{s,t,q,1000}-{T}_{s,t,q,{sz}}\right|$$

3 $${{MBE}}_{{sz}}=\frac{1}{{mnp}}\mathop{\sum }\limits_{q=1}^{m}\,\mathop{\sum }\limits_{t=1}^{n}\,\mathop{\sum }\limits_{s=1}^{p}{\omega }_{s}^{{\prime} }({T}_{s,t,q,1000}-{T}_{s,t,q,{sz}})$$

where MAEs and MBEs are calculated as area-weighted (${\omega }_{s}^{{\prime} }$) means for each sample size sz based on temperature anomalies T for each quantile q, each year t, and each grid point s. As expected, the MBE depends mainly on the number of ESM-specific MESMER calibrations included to produce the emulations, whereas the MAE depends also substantially on the number of realizations (Fig. 4). For 10 stochastic MESMER realizations we find an MAE of 0.049 °C. Increasing the number of stochastic MESMER realizations from 10 to 20 decreases the MAE by less than 0.015 °C.

**Fig. 5:**
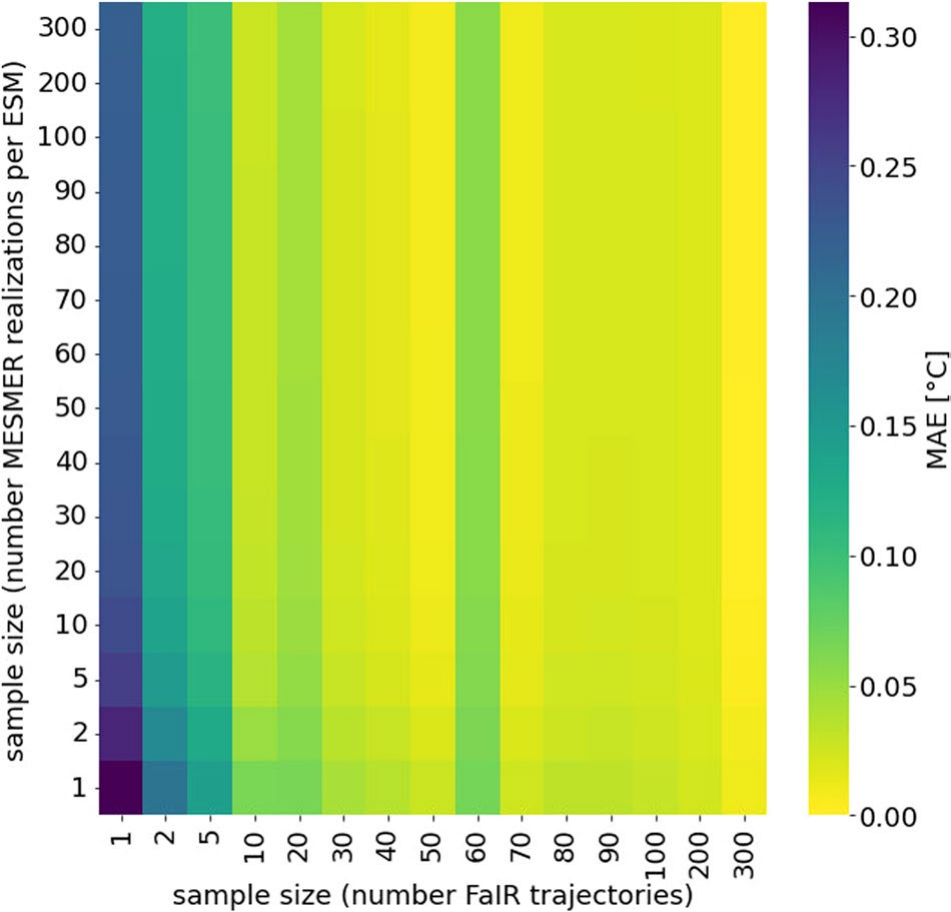
MAEs between quantiles calculated for a varying number of FaIR GMT trajectories and MESMER realizations per ESM.

Third, we created a large number of MESMER emulations from 300 FaIR GMT trajectories (again selected from the “ssp534-over” scenario) and 300 stochastic realizations for each ESM-specific MESMER calibration, for a total of 90’000 emulations. Instead of using data for the entire geographic domain, we randomly selected 20 grid points to increase computational efficiency. Again, we calculate quantiles for each of the selected grid-points and compare those based on all 90’000 emulations (as a reference) with those based on a smaller number of emulations. Averaged over the selected grid points, the results show that the MAE is well below 0.05 °C when more than 100 FaIR trajectories and at least one MESMER realization per ESM-specific calibration for each of these trajectories are included (Fig. 5).

Based on these experiments with different sampling strategies and different sample size, we decided to generate the final ensemble of emulations^21^ in the following way: First, we used balanced clustering to select 100 FaIR GMT trajectories for each of the 10 emission scenarios. Second, we created MESMER emulations based on each of the selected 100 FaIR GMT trajectories. To do this, we used all 25 available ESM-specific parameter sets and for each of these sets we produced 10 stochastic realizations, resulting in a total of 25’000 emulations. The selected number of MESMER and FaIR realizations substantially minimizes potential errors and biases. Further increasing the number of realizations will lead to a considerable increase in storage requirements, while only marginal gains in terms of error reduction can be expected.

## Data Records

**Fig. 6:**
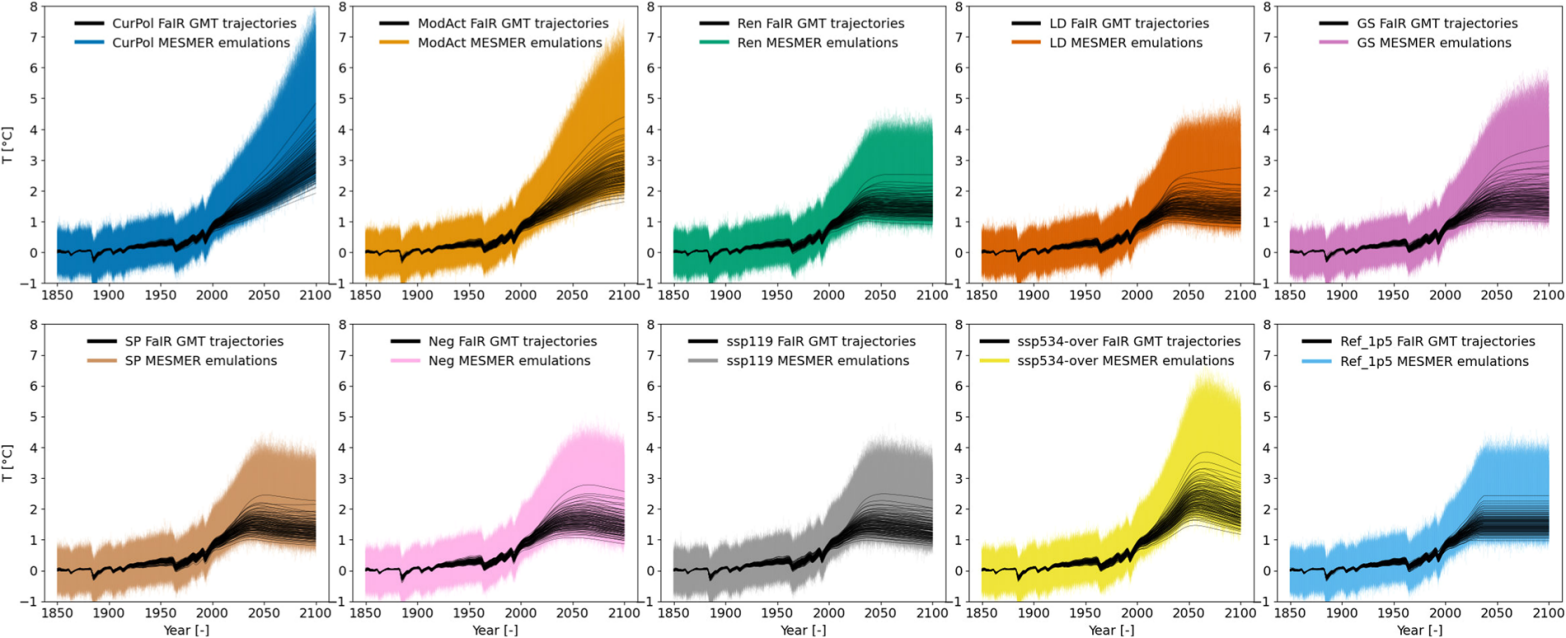
Selected FaIR GMT trajectories showing temperature anomalies for each scenario (100 for each scenario) in black and spatially averaged MESMER emulations (i.e. averages of emulated land temperature anomalies without Antarctica) in color.

The produced annual mean temperature anomaly data is available for each year between 1850 and 2100 and for every land grid-point at a spatial resolution of 2.5°^21^. The data spans four additional dimensions, which are (i) the number of emission scenarios (10), (ii) the number of FaIR realizations (100), (iii) the number of ESM-specific simulations used in the calibration process (25) and (iv) the number of stochastic MESMER realizations per ESM-specific calibration (10, Table 1). Thus, 25’000 emulations are available for every emission scenario, and the distribution in temperature for each grid-point and each year can be estimated from these 25000 temperature values (Fig. 6).

The dataset is openly available (10.3929/ethz-b-000669765, Schwaab, *et al*.^21^). It is structured into 10 folders, one for each scenario. The folders can be downloaded as zip-files and have an approximate size of 140 GB. They are named according to the 10 scenarios (Table 1). Each folder contains 100 NetCDF files, i.e., one file per FaIR realization. Each file has a size of 1.4 GB and is named as follows:

<emission scenario>_<FaIR realization id>.nc

An example of this is the file “ssp534-over_78.nc”, which contains emulations based on the overshoot scenario “ssp534-over” and of a selected FaIR trajectory (id: 78) of this scenario. The NetCDF files unfold along 7 dimensions, which are listed in the following together with their length given in brackets: mesmer_esm_calibration (25), fair_scenario (1), run_id (1), realization (10), year (251), lat (56) and lon (143). While the fair scenario and its realization id (i.e. run_id) are determined by the filename, we kept them as dimensions to facilitate combination of the data and to have a complete description within each file. It should be noted that the dimension “realization” refers to the stochastic MESMER realization and not to the FaIR realization, which is denoted as run_id.

**Fig. 7:**
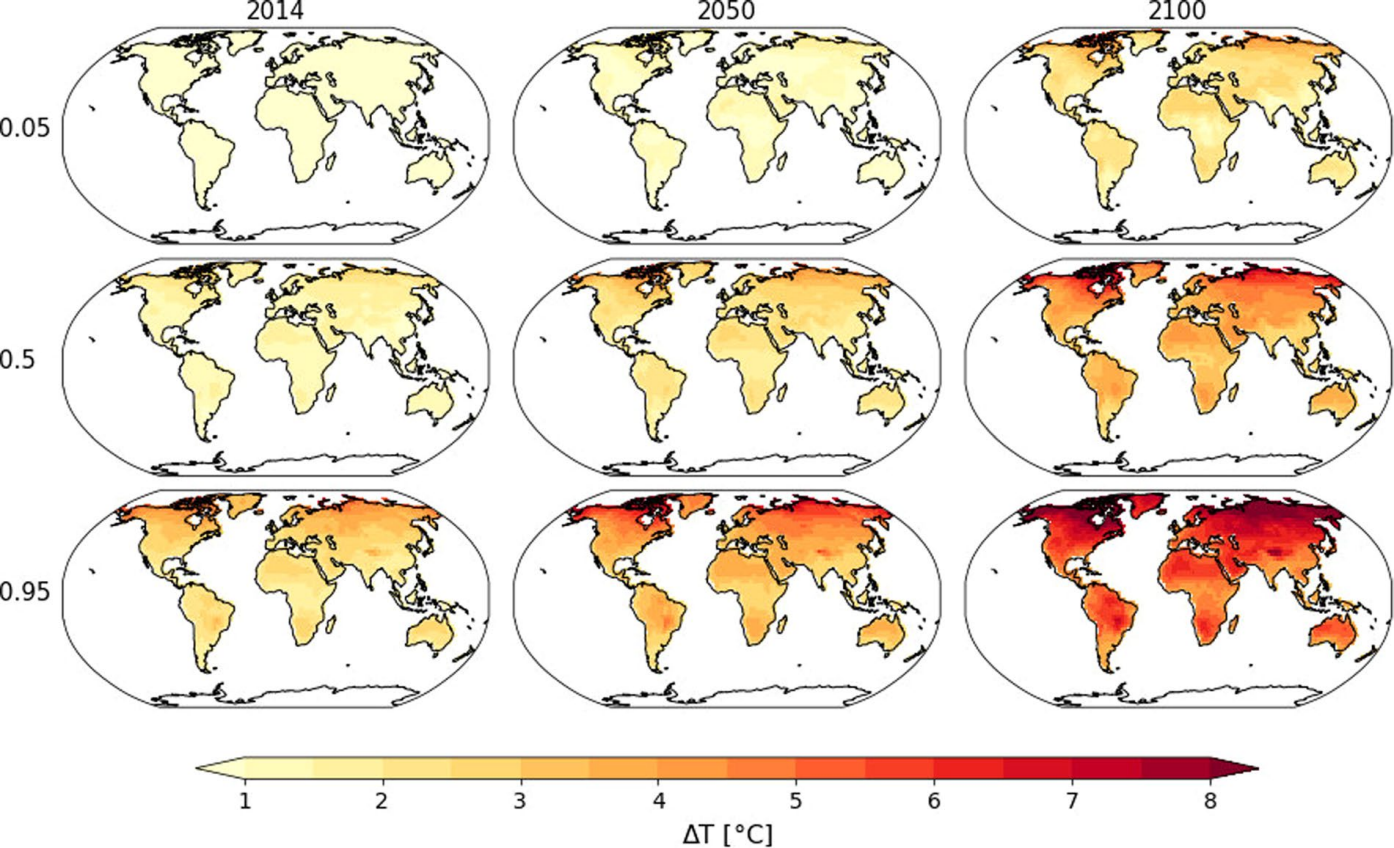
Examples of quantiles of temperature anomalies derived from all emulations of the CurPol scenario.

Based on the emulated data, we also calculated temperature quantiles. These are provided in one tar-file containing 10 NetCDF files, one for each scenario with a file size of 116 MB. For example, “ssp534-over.nc” contains the quantiles (0.05, 0.01, …, 0.9, 0.95 as well as 0.66 and 0.98) for the “ssp534-over” scenario (Fig. 7). The structure of the file is given by its four dimensions: year (251), quantile (21), lat (56) and lon (143). In addition, the reference temperature calculated for the period of 1850–1900 is provided for each ESM in the file “reference_data_1850_1900.tar” (0.5 MB). These files include three dimensions: ESM (25), lat (56) and lon (143). This temperature can be added to the emulated data to obtain absolute temperatures. However, depending on the application, absolute temperature data should not be used before proper bias correction has been applied (see also the section Usage Notes). The unit of the reference temperature and the temperature anomalies is °C.

## Technical Validation

The presented data set is based on the established FaIR and MESMER emulators, which have been extensively validated^9–11^. MESMER parameters (calibrated based on CMIP6 data) that define the behavior of the MESMER emulator are visualized to facilitate the interpretation of differences between ESM-specific MESMER emulations (Figures S4–S6).

For the technical validation of the data we preformed two tests. The first test was to ensure that the generated emulations behave as expected and according to the MESMER parameters. This test included the following steps: First, the MESMER emulations for a specific scenario were averaged across all grid cells and across all FaIR and MESMER realizations. Second, MESMER intercepts were averaged across space (resulting in an average intercept per ESM-specific calibration) and subtracted from the temperature anomaly time series generated in the first step (specific to each emission scenario and ESM-specific MESMER calibration). Third, the temperature time series (resulting from step 2) are divided by the mean of the FaIR GMT trajectories for the selected scenario. The generated ratio of the time series is approximately constant for all emission scenarios (exemplified for the CurPol emission scenario in Figure S7) and close to the average of the pattern scaling parameters (i.e. the local regression coefficients, Figure S8). This is the desired and expected behavior, as the variability components of MESMER center around zero and average out. The remaining signal of the forced response is therefore mainly defined by the local regression coefficients.

As a second test, we analyze whether the data of CMIP6 simulations of overshoot scenarios (available from the public CMIP6 archive: https://esgf-node.llnl.gov/projects/esgf-llnl/) agree with the emulated data that is presented here. We used all CMIP6 simulations ESMs included in Beusch, *et al*.^11^ that provide simulations for ssp534-over (6 ESMs) and ssp119 (7 ESMs).

To facilitate the comparison, we first calculate the temperature anomalies in the CMIP6 simulations with respect to the reference period (1850 – 1900). After that we compare the spatial pattern of the temperature anomalies in the CMIP6 simulations with those from the emulations. For that we calculate mean temperatures from 2014–2100, 2041–2060 and 2081–2100 averaged across initial-condition ensemble members available for each ESM in CMIP6 and across all emulations available for that specific ESM. The results show that there is generally a good agreement between the spatial patterns of the temperature anomalies derived from the CMIP6 simulations and the ones derived from the emulations (Figures S9–S14). Correlations between simulations and emulations are higher than 0.96 for all tested ESMs when comparing mean temperatures of 2014–2100. When averaging temperatures for 2081–2100, there is still a good agreement, however, with the exception of the closely related models CESM2-WACCM and NorESM2-LM in ssp534-over. For these two models the correlation drops below 0.9. There may be two main reasons for this. First, for NorESM2-LM there is only one initial-condition ensemble available. This means, for example, that the spatially averaged fields for NorESM2-LM are slightly less smooth than the ones coming from the 1000 emulations available for NorESM2-LM. A second reason for this difference may be that the patterns of the temperature change in overshoot scenarios are uncertain and can change after peak warming^27^. This is a feature that MESMER will not fully capture since parameters are calibrated for several scenarios. The CMIP6 simulations and the emulations can only be compared in their spatial patterns and not in terms of absolute temperature anomalies. The latter is mostly determined by the FaIR emulations, which are not specific to each ESM. Only the MESMER emulations that determine the spatial pattern of the anomaly are ESM-specific.

In a second step, we calculate the global mean temperature timeseries of the available CMIP6 simulations and all emulations. Here it should also be noted that a comparison of the global mean temperature timeseries is not possible on an ESM-specific basis. However, it is possible to show that the temperature timeseries of the CMIP6 data lie within the range of the emulations and that their trajectories are very similar (Figure S15).

## Usage Notes

A combination of FaIR and MESMER allows for the translation of any greenhouse gas emission scenario into spatially resolved annual mean temperature anomalies. Here, we used this combination to produce spatially resolved emulated annual temperature projections for specific overshoot pathways. The data can be used to evaluate the impacts of a range of overshoot scenarios and to critically assess climate mitigation and adaptation efforts.

We outlined in which way and how many emulations were produced to approximate the temperature quantiles at the grid-point level reasonably well. However, it is important to note that temperature quantiles were used for evaluation purposes only and should not be used in all downstream applications of the emulated data. In many downstream applications, such as the estimation of socio-economic and environmental impacts of temperature changes, a larger number of individual emulations should be used to ensure proper uncertainty propagation. The adequate choice of number of emulations and method of subsampling ultimately depends on the purpose of the data and acceptable trade-offs between precision and data storage capacity. If refined uncertainty estimates are required, it could be an option to run MESMER for a limited number of time-steps and only for specific regions, thereby reducing the amount of data that is generated.

To reduce the number of emulations, one of the strategies outlined in this study was to cluster the FaIR data. The clustering helped to preserve some key features of the ensemble. The clustering approach could be extended by applying it to the spatially resolved emulations. This step would be computationally demanding but might help to further reduce the number of emulations (e.g. by using similar features in different ESMs) while preserving their main spatio-temporal features.

By controlling the number of emulations, it is possible to replicate the uncertainty that FaIR and MESMER can represent by design. Uncertainties and biases that arise from the parameterization and functionality of the emulators can mostly not be addressed by using a specific sampling approach and by increasing the number of emulations. Such uncertainties include, e.g., that the MESMER data is slightly underdispersive at the regional scale compared to ESM data^10^. In addition, it should be mentioned that we rely on linear pattern-scaling. This approach has been shown to work very well^11^, but may not fully account for non-linearities in the climate system when going from a transient to an equilibrium climate^28,29^. While Beusch, *et al*.^11^ have demonstrated that a more flexible pattern-scaling – allowing for additional and non-linear predictors – does not perform significantly better than a linear approach, it may be of certain relevance for overshoot scenarios that rapidly converge to an equilibrium state.

## Supplementary information

Supplementary Information

## Data Availability

The code for both emulators is openly available. The code for MESMER is available under https://github.com/MESMER-group/mesmer and the code for FaIR is available under: https://docs.fairmodel.net/en/latest/.
