# Supplementary material for: Spatially resolved emulated annual temperature projections for overshoot pathways
Source: Sci Data. 2024 Nov 21;11:1262. doi: 10.1038/s41597-024-04122-1 (PMC11582622; doi:10.1038/s41597-024-04122-1)
Supplement: Supplementary file 1 — Supplementary Information [file 41597_2024_4122_MOESM1_ESM.docx]

**Supplementary Information**

Table S 1: Scenarios simulated with FaIR. Adapted from Lamboll et al. (2022).

| **#** | **Scenario name** | **Start/End** | **Start scenario** | **Description of modifications** |
| --- | --- | --- | --- | --- |
| 1 | CurPol | 1850/2100 | CurPol | Original scenario from (NGFS, 2020) obtained from AR6 WG3 scenario database (Byers *et al.*, 2022) |
| 2 | ModAct | 1850/2100 | ModAct | Original scenario from (Riahi *et al.*, 2021) obtained from AR6 WG3 scenario database |
| 3 | GS | 1850/2100 | GS | Original scenario from (van Soest et al., 2021) obtained from AR6 WG3 scenario database |
| 4 | NEG | 1850/2100 | NEG | Original scenario from (GNFS, 2020) obtained from AR6 WG3 scenario database |
| 5 | REN | 1850/2100 | REN | Original scenario from (Luderer et al., 2021) obtained from AR6 WG3 scenario database |
| 6 | LD | 1850/2100 | LD | Original scenario from (Grubler et al., 2018) obtained from AR6 WG3 scenario database |
| 7 | SP | 1850/2100 | SP | Original scenario from (Soergel et al., 2021) obtained from AR6 WG3 scenario database |
| 8 | Ref_1p5 | 1850/2300 | LD | Follow LD until the median global surface temperature increase reaches 1.5°C, and keep the entire surface temperature distribution constant thereafter |
| 9 | SSP5-3.4-OS | 1850/2300 | SSP5-3.4-OS | Extension of (O’Neill et al., 2016) from RCMIP (Meinshausen et al., 2020) |
| 10 | SSP1-1.9 | 1850/2300 | SSP1-1.9 | Extension of (Rogelj et al., 2018) from RCMIP |

Table S 2: Description of policy and socioeconomic assumptions behind the scenarios simulated with FaIR. Adapted from Lamboll et al. (2022).

| **Scenario** | **Description** |
| --- | --- |
| CurPol | Current policy pathway. This pathway explores the consequences of continuing along the path of implemented climate policies in 2020 with only mild strengthening after that. The scenario illustrates the outcomes of many scenarios in the literature that project the outcomes of current policies. |
| ModAct | Moderate action pathway. This pathway explores the impact of countries sticking to their Nationally Determined Contributions (NDCs) as stated in 2020. These are often more ambitious than currently implemented policies, but for most countries do not ratchet up very rapidly. Similar levels of mitigation effort are expected going forwards. |
| GS | Gradual strengthening pathway. Energy demand is reduced in the 2030s and the transition to variable renewable energy accelerates then too. Renewable energy never forces out all fossil fuel use – carbon dioxide is captured from the air and buried instead, along with reforestation. |
| Neg | Pathway with highest negative emissions (carbon-removing technology). Fossil fuel use decreases slowly, replaced about equally by variable renewable energy and biofuels. The carbon from burning the biofuels is captured and buried, offsetting the continued fossil fuel use. |
| Ren | Renewable pathway. Energy demand is reduced rapidly in the short-term, though grows later. Growth in renewables is very rapid and squeezes out most all other types of energy. Some biofuel is used to balance renewable variability, and the emissions from this are captured and buried. |
| LD | Demand-limiting pathway. Energy demand is massively reduced by implementing energy efficient lifestyles and design, and kept low throughout the century. Renewable energy grows and gradually forces out fossil fuels. |
| SP | This ‘Shifting Pathways’ scenario explores how a broader shift towards sustainable development can be combined with climate policies consistent with keeping warming to 1.5˚C. Energy demand is reduced over time, while renewable energy use grows, squeezing out fossil fuel use. |
| SSP1-1.9 | Renewable energy is deployed rapidly. Energy demand is also limited rapidly. There is general focus on sustainability, but also a significant increase in the amount of biofuel use, with the carbon released by this captured and stored (negative emissions). |
| SSP5-3.4-OS | Carbon emissions rise at an incredibly fast rate in the short term. Then, around 2040 they decline extremely rapidly through the massive use of negative emissions technologies (for example capturing carbon from biofuel burning or directly from the air, and burying it). This pathway was designed to test the sensitivity of the Earth System to such extreme changes in emissions. |
| Ref_1.5C | Temperatures rise as in the demand-limiting case (LD) until the global average reaches 1.5°C. The temperature is then held constant. This scenario is a simple thought-experiment not driven by economic or climatic considerations. |


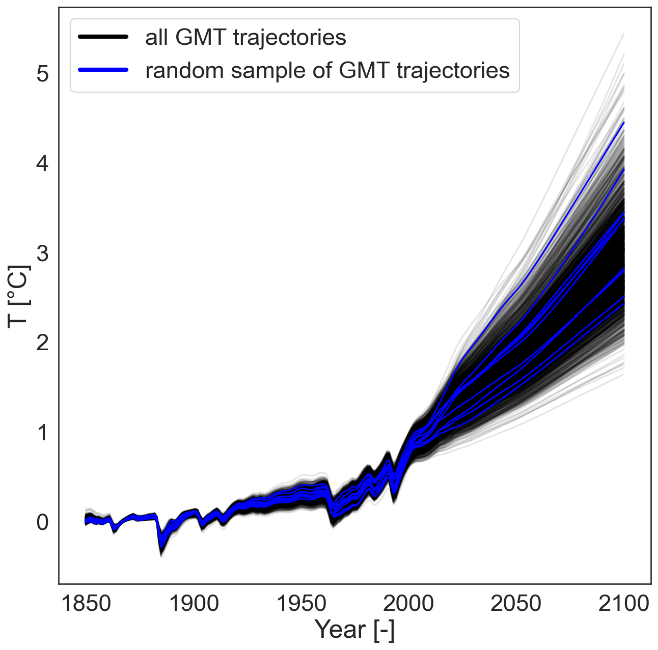

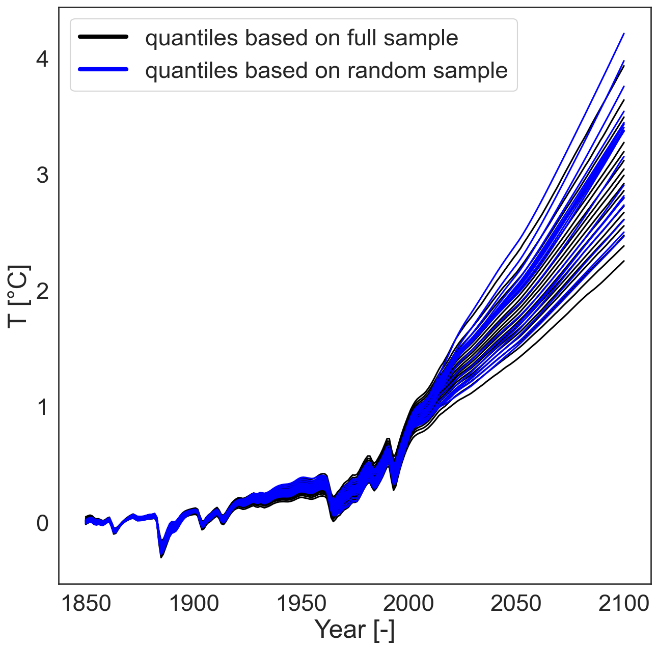


Figure S 1: Left: Example of all and some randomly selected GMT anomaly trajectories. Right: Quantiles (0.05-0.95) calculated when including all GMT anomaly trajectories and when including only the random sample of GMT anomaly trajectories.


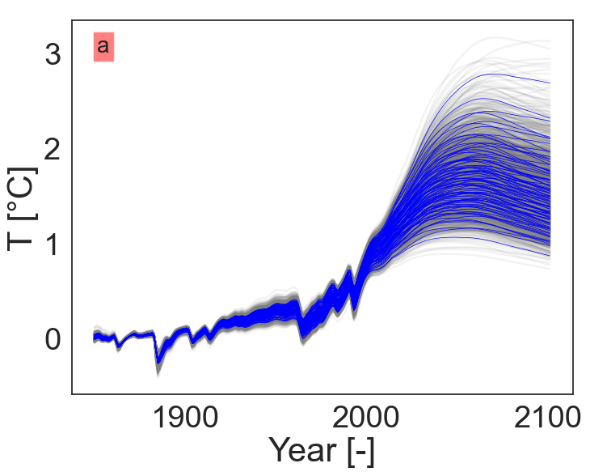

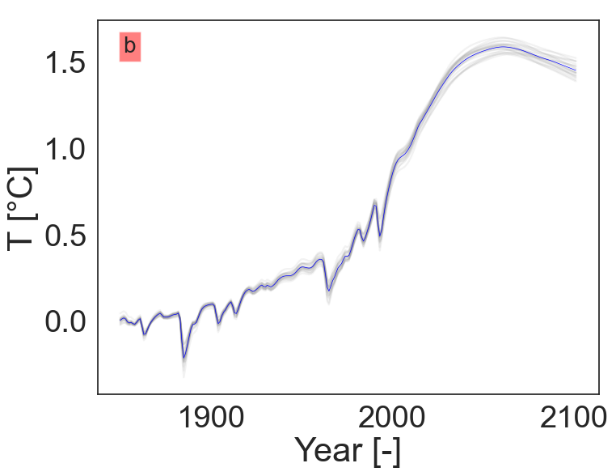


Figure S 2: a) Cluster centers that were derived by the balanced clustering approach are shown in blue and all GMT anomaly trajectories are shown in grey. b) Depiction of a single cluster together (grey trajectories) together with a single anomaly trajectory (blue) that is closest to the center of this specific cluster.


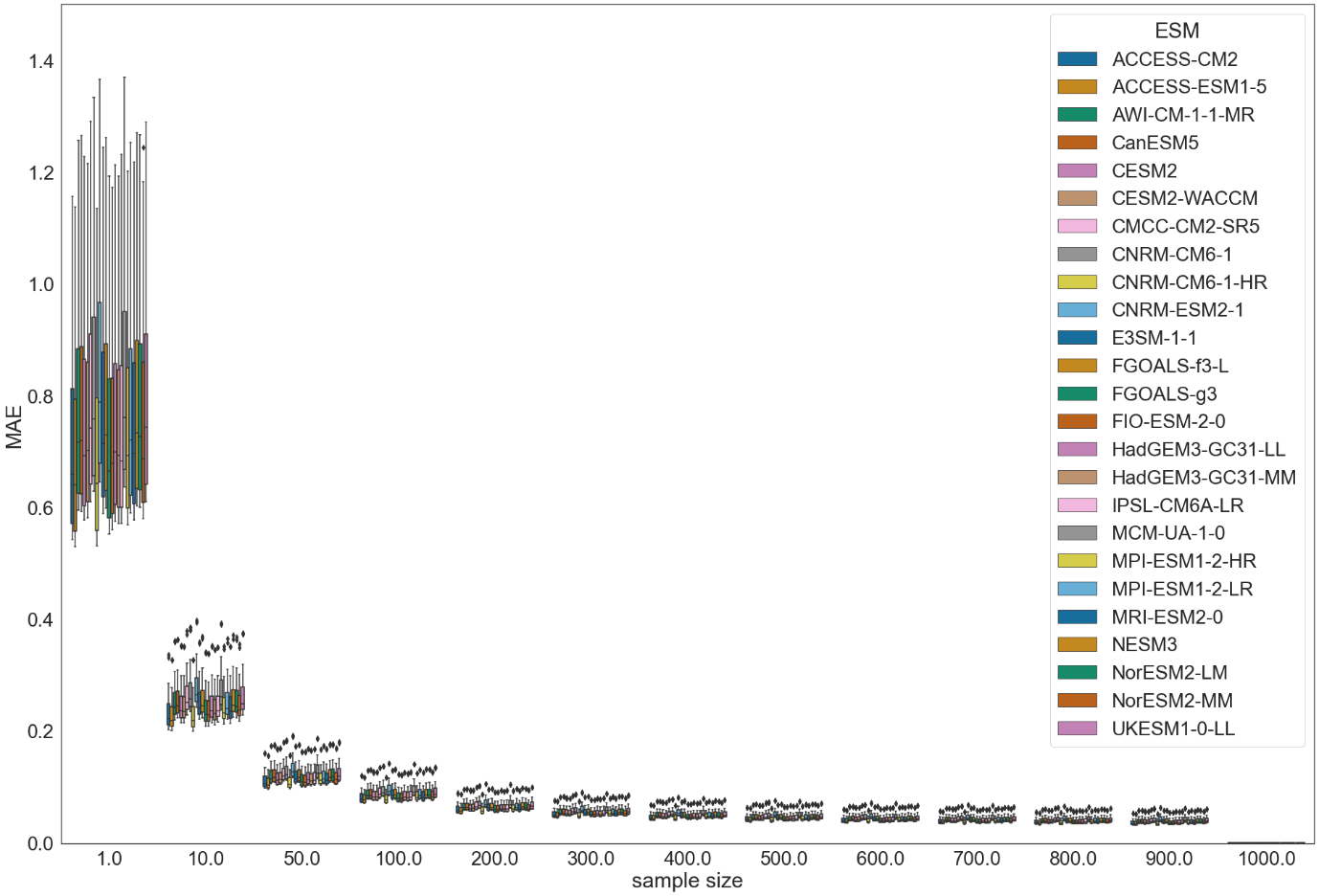


Figure S 3: MAEs calculated between quantiles of varying number of MESMER realizations for each grid point (e.g. the MAE for sample size 100 indicates the MAE calculated between quantiles estimated based on 1000 realizations and quantiles based on 100 emulations).


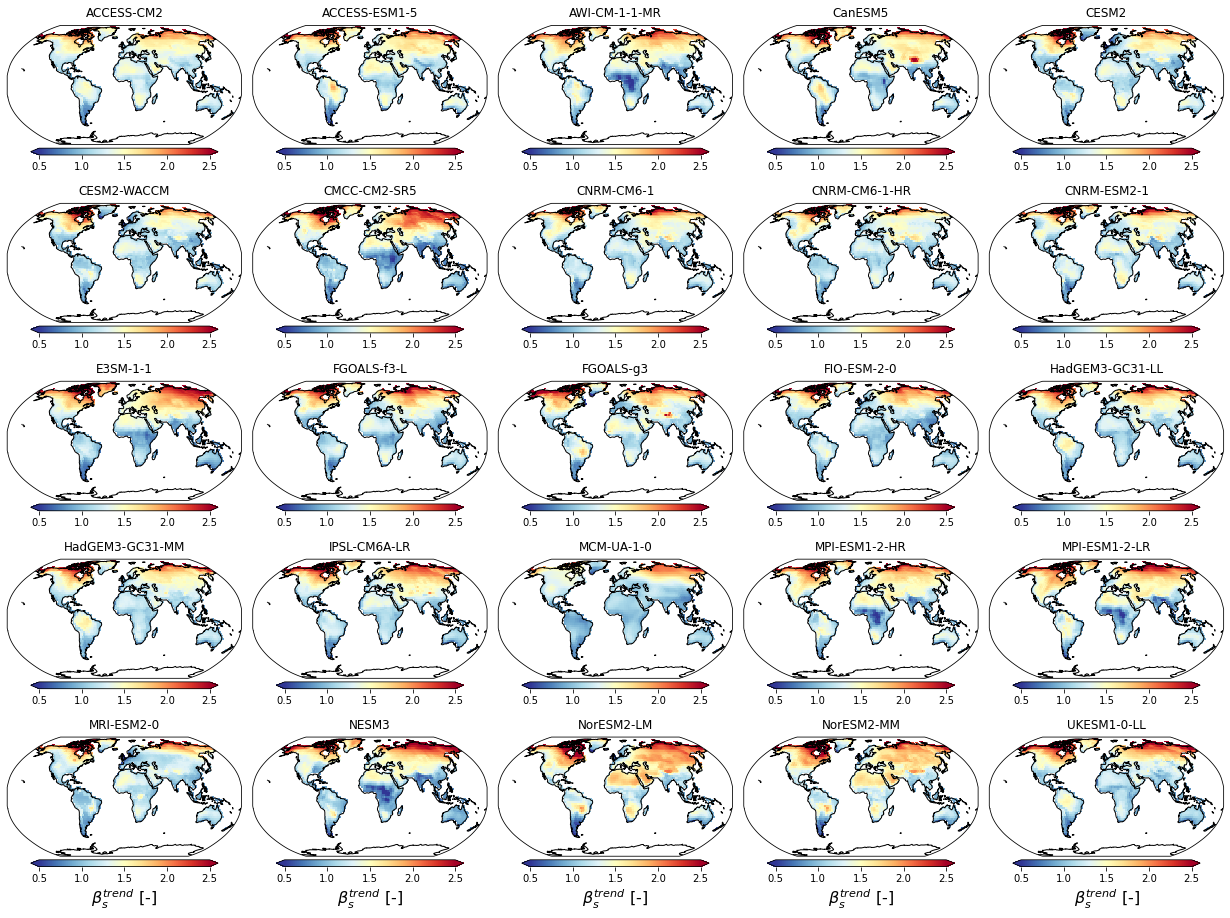


Figure S 4: Spatial distribution of the regression coefficients that link the forced global to the local temperature response in MESMER.


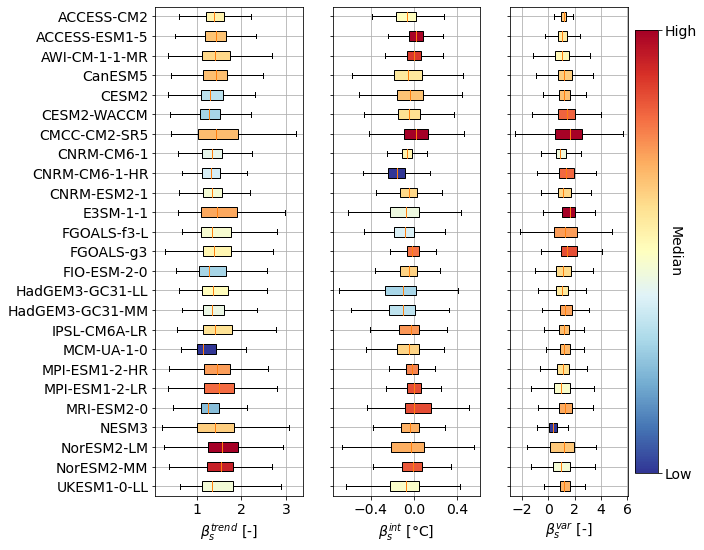


Figure S 5: MESMER parameters essential to the local response module of MESMER (Beusch et al., 2020). All parameters were calibrated using CMIP6 data. Outliers have been removed to ease interpretation. The boxes extend from the first quartile (Q1) to the third quartile (Q3), with a line at the median. The whiskers extend from the boxes to the farthest data point lying within 1.5x the inter-quartile range (IQR) from the boxes. The boxes are colored according to their median values from low median values (dark blue) to high median values (dark red).


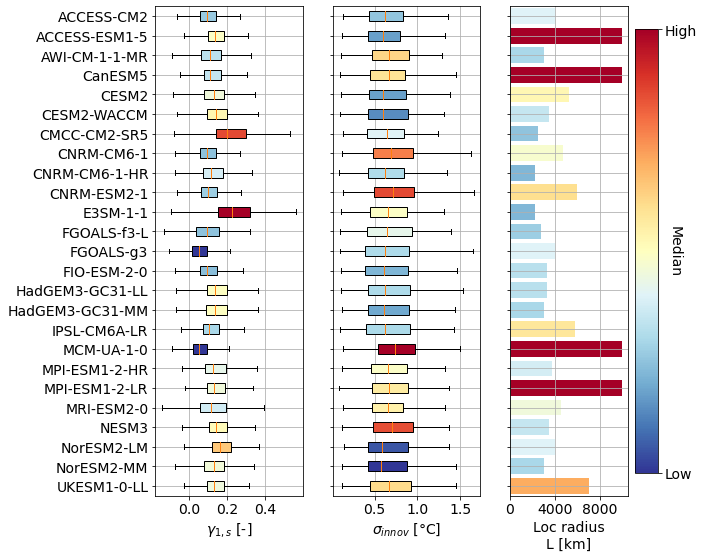


Figure S 6: MESMER parameters indicating properties of the variability module of MESMER (Beusch et al., 2020). All parameters were calibrated using CMIP6 data. Outliers have been removed to ease of interpretation. The boxes extend from the first quartile (Q1) to the third quartile (Q3), with a line at the median. The whiskers extend from the boxes to the farthest data point lying within 1.5x the inter-quartile range (IQR) from the boxes. The boxes are colored according to their median values from low median values (dark blue) to high median values (dark red). The bars indicating the localization radius are colored according to their values ranging from low localization radii (dark blue) to high localization radii (dark red).


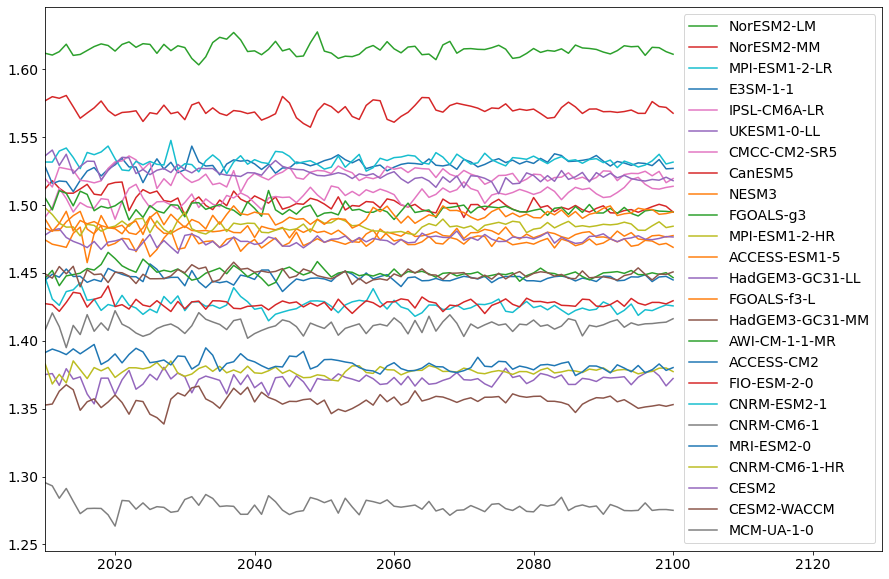


Figure S 7: The time series shown in this figure are the result of dividing the averaged MESMER emulations of the CurPol emission scenario (averaged over space and across 1’000 emulations available for each ESM-specific MESMER calibration) by the averaged FaIR emulations for the CurPol scenario (averaged over all FaIR emulations selected by the clustering process). Since the local forced response is not only based on the local regression coefficient that links global and local forced response, but also on the intercept that is added to derive the local response, the intercept was subtracted from the averaged MESMER emulations before dividing them by the averaged FaIR emulations.


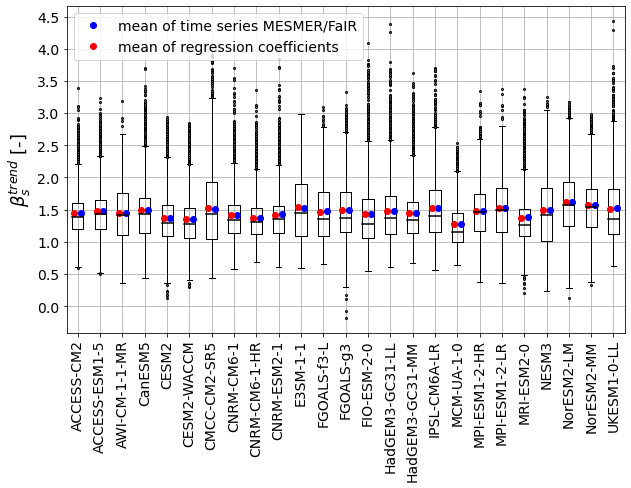


Figure S 8: Boxplots of the regression coefficients together with the mean of the regression coefficients and the mean of the quotient of the averaged MESMER and FaIR time series as shown and described in Figure S 7. The boxes extend from the first quartile (Q1) to the third quartile (Q3), with a line at the median. The whiskers extend from the boxes to the farthest data point lying within 1.5x the inter-quartile range (IQR) from the boxes.


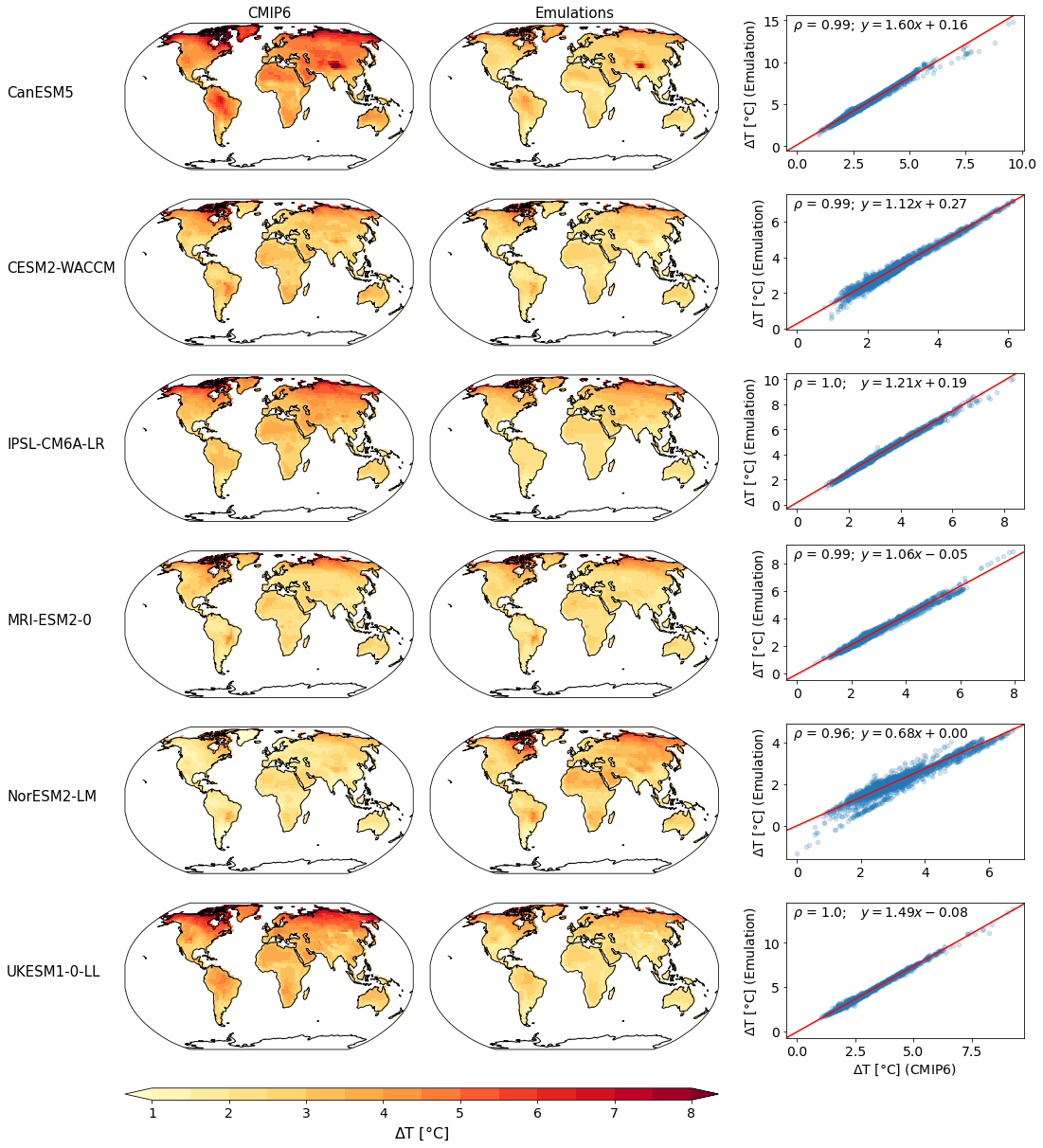


Figure S 9: Maps showing mean temperatures for the scenario ssp534-over of each ESM from 2014-2100. For the CMIP6 simulations the data is averaged across all initial-condition ensemble members available for each specific ESM (left column). For the emulations of each ESM available in CMIP6 the data has been averaged across all available emulations (central column). In the right column are scatterplots that illustrate the correlation between CMIP6 simulations and the emulations. In these scatterplots we show the Pearson correlation coefficient (*ρ) and a regression line together with its equation.*


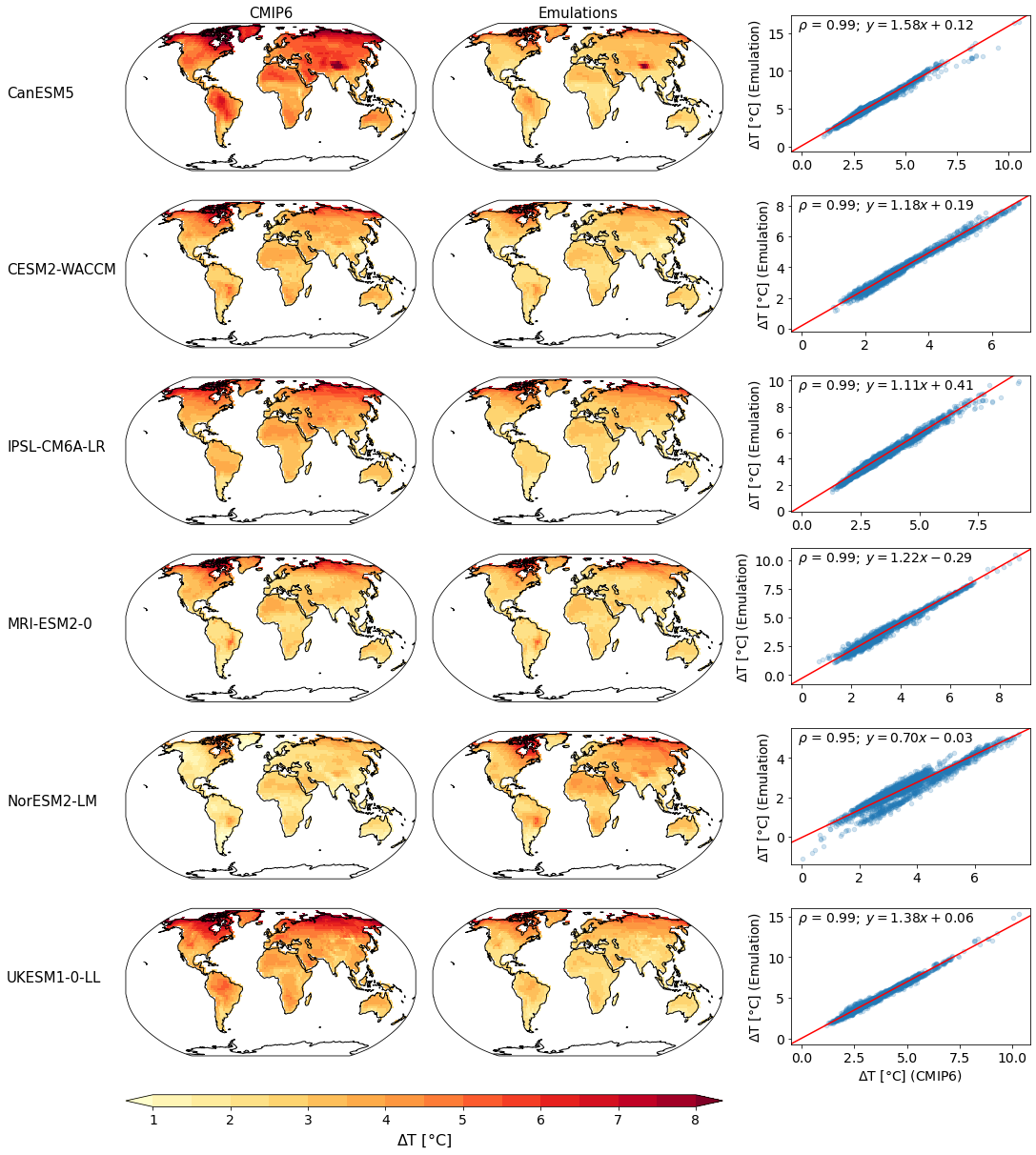


Figure S 10: Same as Figure S 9, but mean temperatures of 2041-2060.


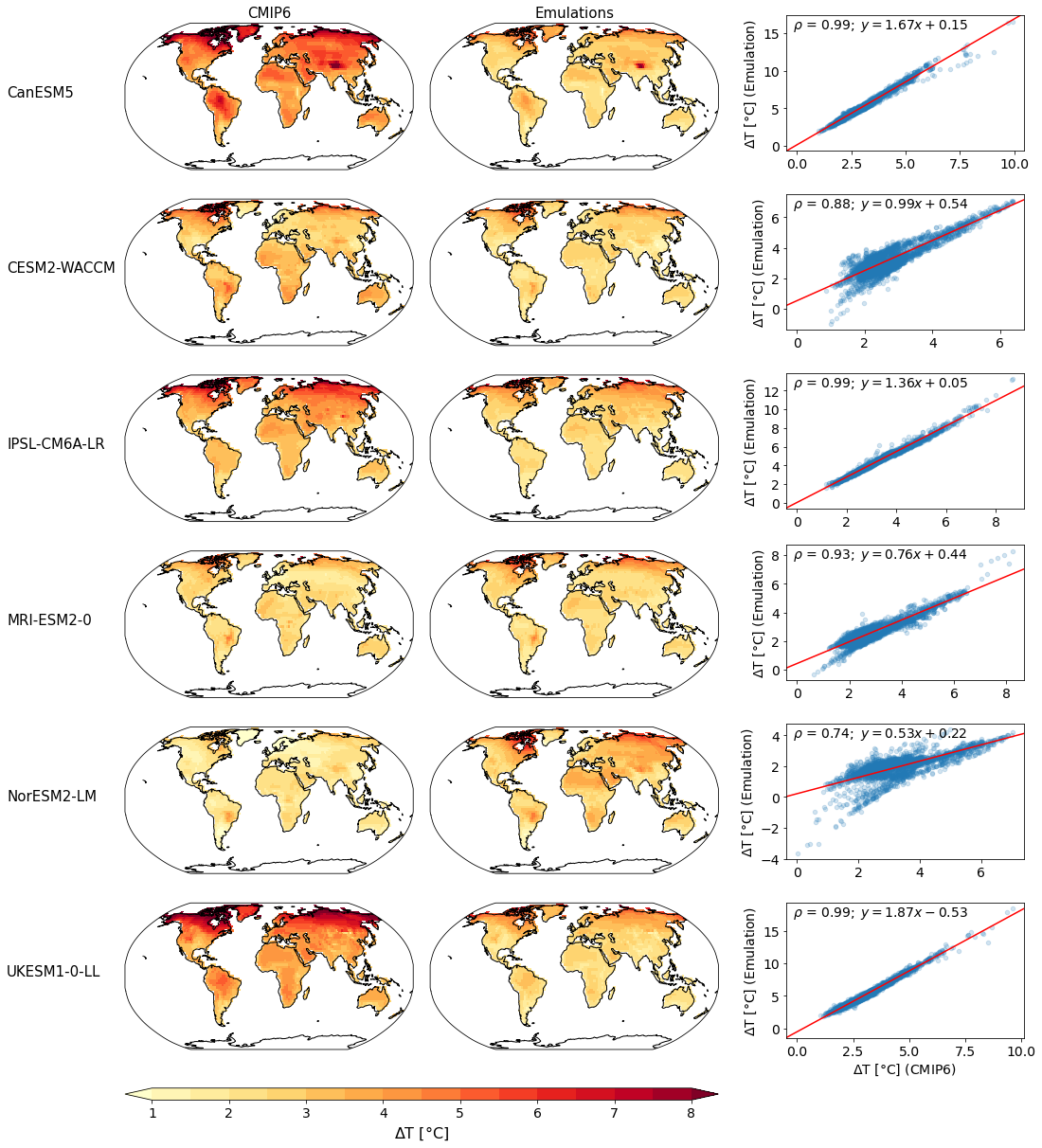


Figure S 11: Same as Figure S 9/S10, but mean temperatures of 2081-2100.


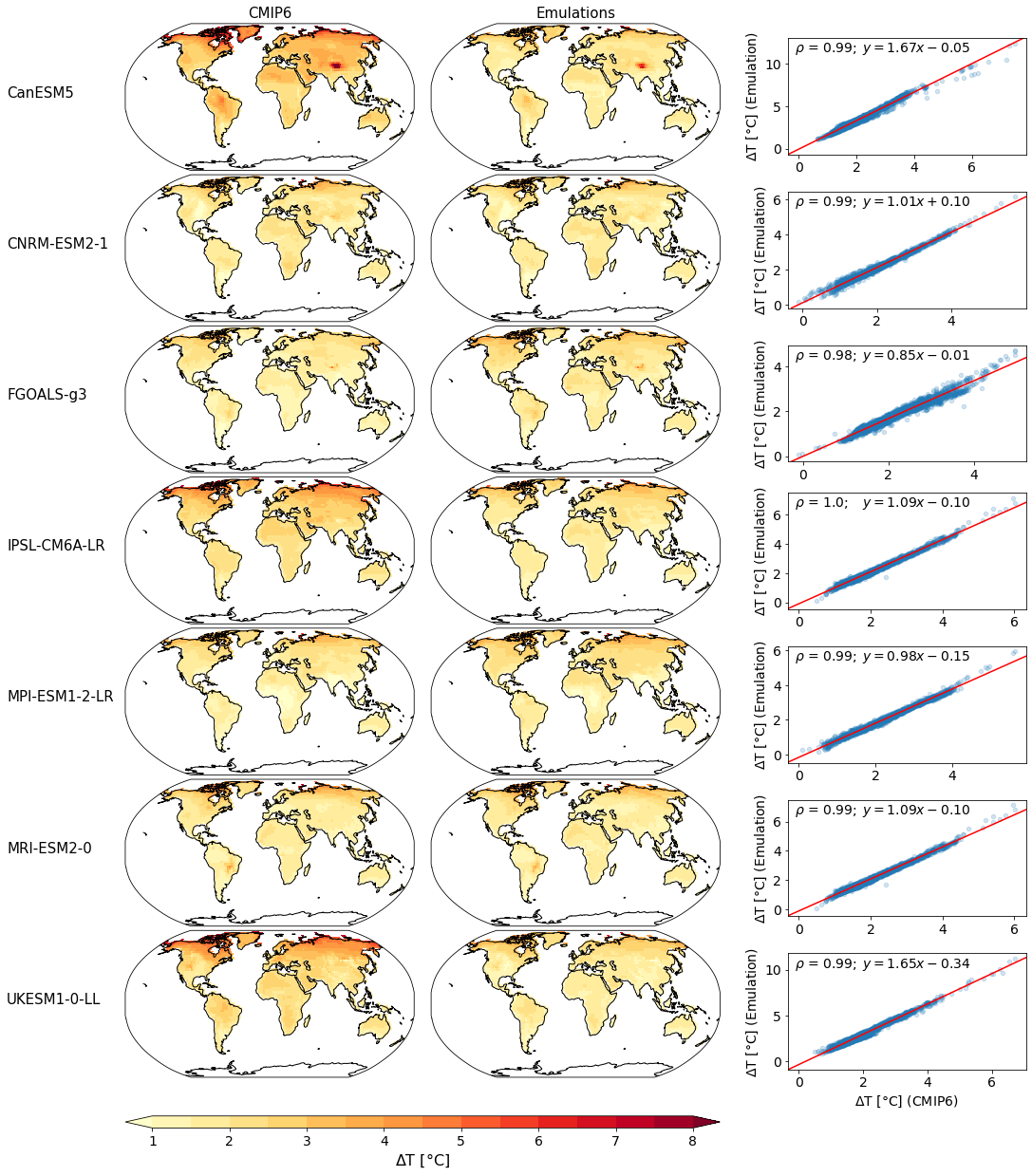


Figure S 12: Maps showing mean temperatures for the scenario ssp119 of each ESM from 2014-2100. For the CMIP6 simulations the data is averaged across all initial-condition ensemble members available for each specific ESM (left column). For the emulations of each ESM available in CMIP6 the data has been averaged across all available emulations (central column). In the right column are scatterplots that illustrate the correlation between CMIP6 simulations and the emulations. In these scatterplots we show the Pearson correlation coefficient (*ρ) and a regression line together with its equation.*


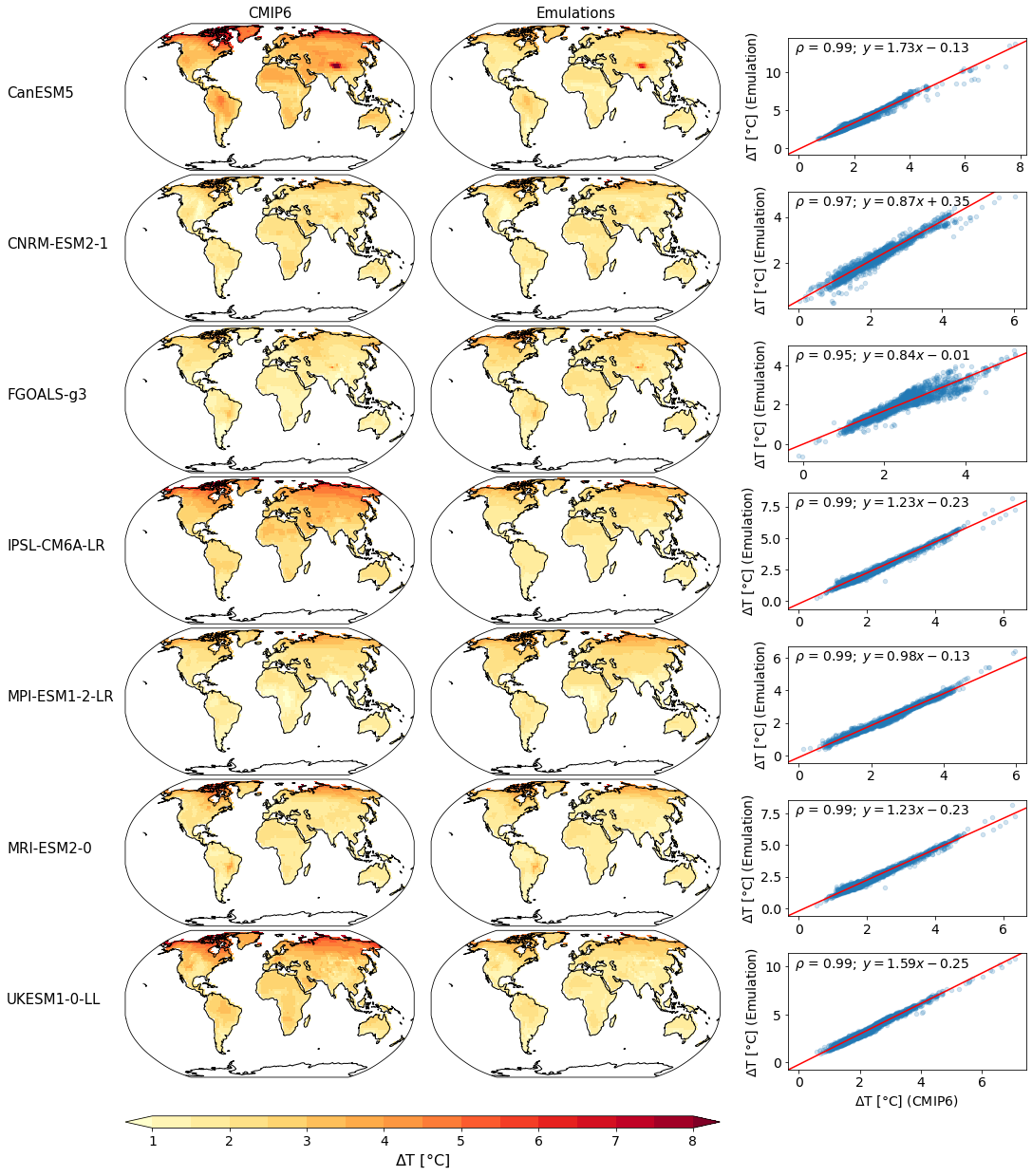
Figure S 13: Same as Figure S 12, but mean temperatures of 2041-2060.
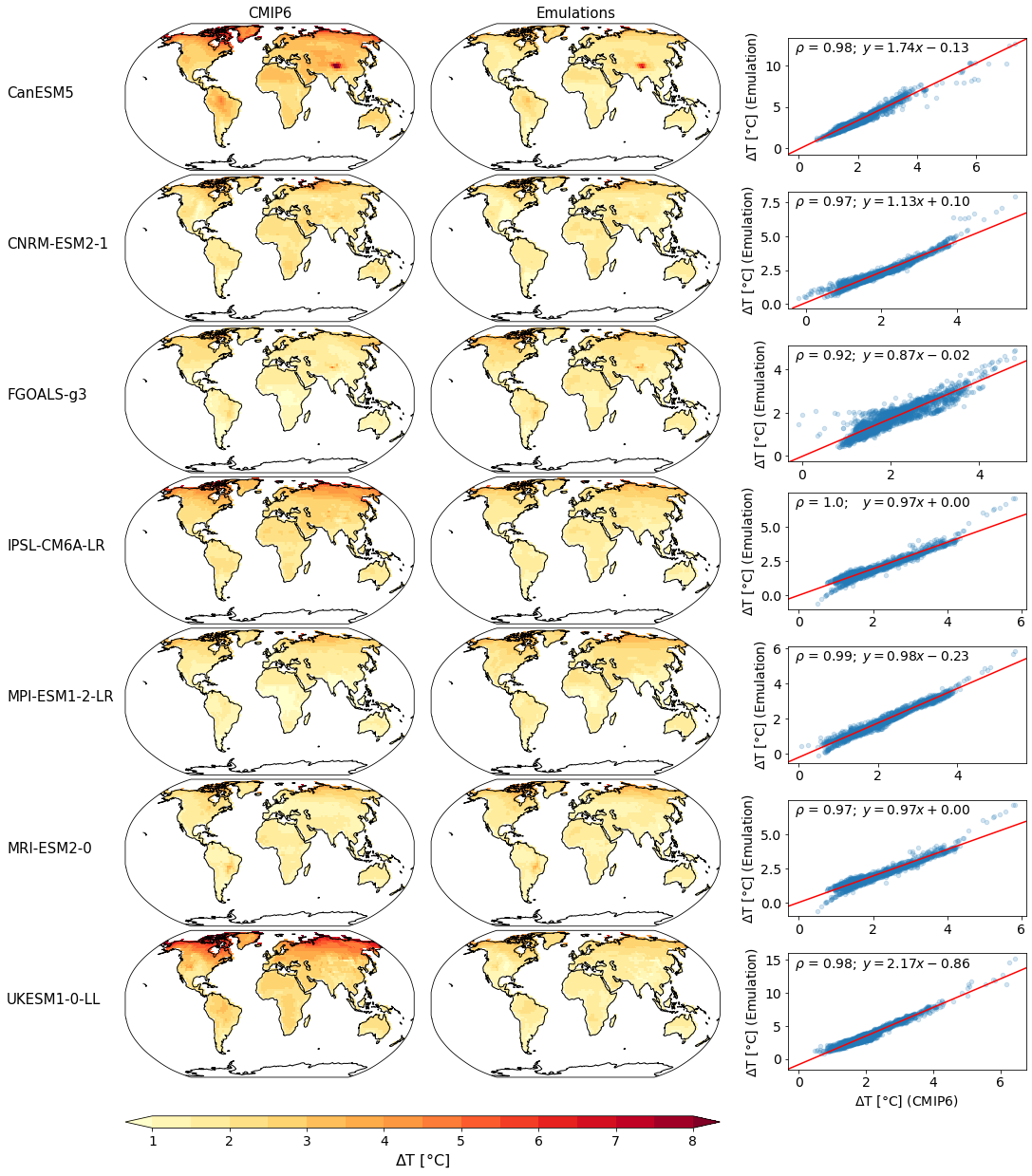


Figure S 14: Same as Figure S 12/13, but mean temperatures of 2081-2100.


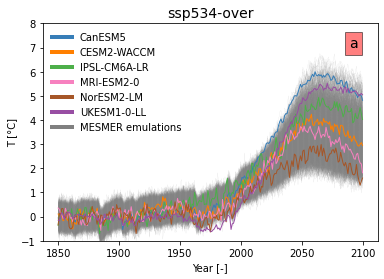

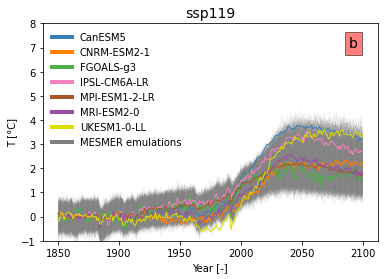


Figure S 15: Global mean temperature anomaly timeseries of the available CMIP6 simulations and all emulations. a) Timeseries for ssp534-over. b) Timeseries for ssp119.

BEUSCH, L., GUDMUNDSSON, L. & SENEVIRATNE, S. I. 2020. Emulating Earth system model temperatures with MESMER: from global mean temperature trajectories to grid-point-level realizations on land. *Earth Syst. Dynam.,* 11**,** 139-159.

LAMBOLL, R., ROGELJ, J. & SCHLEUSSNER, C.-F. 2022. A guide to scenarios for the PROVIDE project. *Earth and Space Science Open Archive***,** 14.
